# Manipulating or Superseding Host Recombination Functions: A Dilemma That Shapes Phage Evolvability

**DOI:** 10.1371/journal.pgen.1003825

**Published:** 2013-09-26

**Authors:** Louis-Marie Bobay, Marie Touchon, Eduardo P. C. Rocha

**Affiliations:** 1 Microbial Evolutionary Genomics, Institut Pasteur, Paris, France; 2 CNRS, UMR3525, Paris, France; 3 Université Pierre et Marie Curie, Cellule Pasteur UPMC, Paris, France; Universidad de Sevilla, Spain

## Abstract

Phages, like many parasites, tend to have small genomes and may encode autonomous functions or manipulate those of their hosts'. Recombination functions are essential for phage replication and diversification. They are also nearly ubiquitous in bacteria. The *E. coli* genome encodes many copies of an octamer (Chi) motif that upon recognition by RecBCD favors repair of double strand breaks by homologous recombination. This might allow self from non-self discrimination because RecBCD degrades DNA lacking Chi. Bacteriophage Lambda, an *E. coli* parasite, lacks Chi motifs, but escapes degradation by inhibiting RecBCD and encoding its own autonomous recombination machinery. We found that only half of 275 lambdoid genomes encode recombinases, the remaining relying on the host's machinery. Unexpectedly, we found that some lambdoid phages contain extremely high numbers of Chi motifs concentrated between the phage origin of replication and the packaging site. This suggests a tight association between replication, packaging and RecBCD-mediated recombination in these phages. Indeed, phages lacking recombinases strongly over-represent Chi motifs. Conversely, phages encoding recombinases and inhibiting host recombination machinery select for the absence of Chi motifs. Host and phage recombinases use different mechanisms and the latter are more tolerant to sequence divergence. Accordingly, we show that phages encoding their own recombination machinery have more mosaic genomes resulting from recent recombination events and have more diverse gene repertoires, i.e. larger pan genomes. We discuss the costs and benefits of superseding or manipulating host recombination functions and how this decision shapes phage genome structure and evolvability.

## Author Summary

Bacterial viruses, called bacteriophages, are extremely abundant in the biosphere. They have key roles in the regulation of bacterial populations and in the diversification of bacterial genomes. Among these viruses, lambdoid phages are very abundant in enterobacteria and exchange genetic material very frequently. This latter process is thought to increase phage diversity and therefore facilitate adaptation to hosts. Recombination is also essential for the replication of many lambdoid phages. Lambdoids have been described to encode their own recombination genes and inhibit their hosts'. In this study, we show that lambdoids are split regarding their capacity to encode autonomous recombination functions and that this affects the abundance of recombination-related sequence motifs. Half of the phages encode an autonomous system and inhibit their hosts'. The trade-off between superseding and manipulating the hosts' recombination functions has important consequences. The phages encoding autonomous recombination functions have more diverse gene repertoires and recombine more frequently. Viruses, as many other parasites, have small genomes and depend on their hosts for several housekeeping functions. Hence, they often face trade-offs between supersession and manipulation of molecular machineries. Our results suggest these trade-offs may shape viral gene repertoires, their sequence composition and even influence their evolvability.

## Introduction

Genetic recombination plays key roles in biology. Recombinases are required for essential cellular functions such as repair of stalled or collapsed replication forks, DNA repair and chromosome segregation [1], [2]. Recombination also drives genetic diversification and increases the efficiency of natural selection [3], [4]. Inter-genomic recombination allows horizontal gene transfer between organisms and exchange of sequences between viruses infecting the same cell [5]. Illegitimate and homologous recombination events between bacterial viruses (phages) are frequent and result in strongly mosaic genomes, i.e. genomes with strong internal phylogenetic incongruences [6], but the relative importance of each recombination mechanism remains unclear [7]–[9]. The group of lambdoid phages provides a striking example of this phenomenon. These temperate phages account for more than two thirds of *E. coli* prophages [10], and are extremely diverse from the genetic, structural and physiological point of view. Nevertheless, they all have similar genetic organization and this allows the production of viable hybrids by inter-genomic recombination [11], [12]. Lambdoid genomes are organized in relatively autonomous gene clusters with genes being encoded next to their interactants, i.e. genes encoding an interacting protein or the targeted DNA site [13]. Moreover, the organization of morphogenesis genes strikingly reflects the order of the proteins forming the virion structure, suggesting a direct link between gene order and function or structure within each module [13]. The extent and phylogenetic range of genetic exchange can be very large: lambdoids include phages with different virion structures such as Siphovirus Lambda, Podovirus P22 or Myovirus SfV, showing that recombination blurs the traditional taxonomy (based on virion morphology). Nevertheless, two thirds of the lambdoid phages in *E. coli* are closely related to phage Lambda and display a *Siphoviridae*'s virion structure (Lambda-like elements) [10]. Phages and bacteria are in constant evolutionary arms races [14]. Accordingly, bacterial outer membrane structures that are phage attachment sites evolve very fast because of the selective pressure imposed by phages [15]. Reciprocally, phage proteins involved in attachment to the host cell, such as tail-fiber proteins, evolve fast in response to these changes [16]. Recombination both in the bacteria and in the phage facilitates these diversifying selection processes, accelerating the rate of evolution [17].

**Figure 1:**
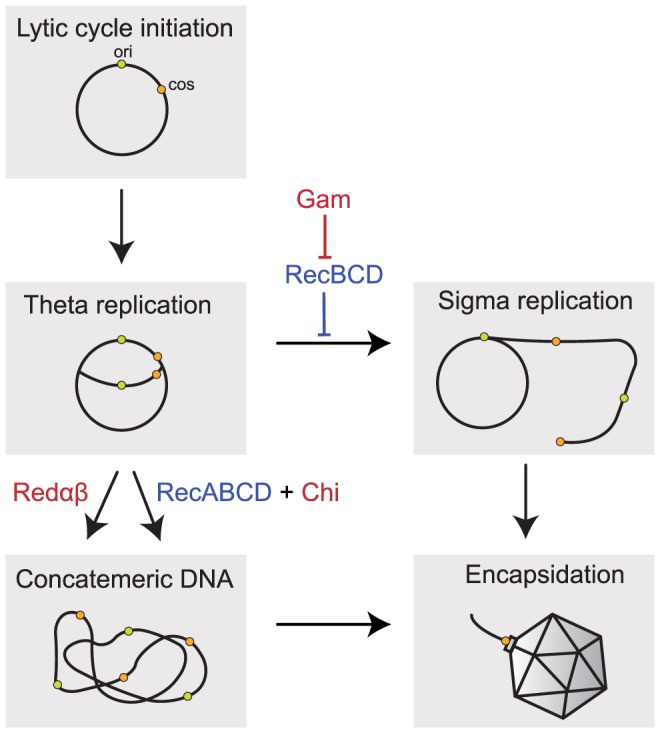
Implication of recombination in the replication of Lambda phage. Packaging of Lambda chromosomes requires concatemeric DNA. The induction of the lytic cycle leads to a number of rounds of theta replication (circle-to-circle). Concatemeric DNA can be formed directly from these newly replicated chromosomes by homologous recombination using the Red pathway, which requires the recombinase Redβ and the exonuclease Redα, or the host RecBCD pathway of recombination specifically enhanced by Chi sites. Concatemers can also be produced by rolling-circle (sigma) replication if the host RecBCD exonuclease is inhibited (e.g. by Gam encoded in Lambda). Concatemers are cleaved by the phage-encoded terminase at their *cos* sites (represented in orange) as they are packaged into the capsid. Lambda encoded sequences are indicated in red, the host encoded genes in blue. *ori* indicates the origin of replication.

Efficient encapsidation of phage Lambda requires concatemeric DNA (reviewed in [18]). These concatemers can be produced by homologous recombination or rolling-circle (sigma) replication (Figure 1). However, rolling-circle replication is inhibited by the exonucleolytic activity of the host RecBCD enzyme from the major homologous recombination pathway [19]. Hence, the phage needs to either block this exonucleolytic activity or produce concatemers by homologous recombination. Phage Lambda encodes its own homologous recombination toolkit under the form of a 3-genes operon [20]: *exo*, *bet* and *gam*, that encode Redα, Redβ and Gam respectively. Redα is a double strand specific 5′ to 3′ exonuclease and Redβ is a recombinase of the Rad52 superfamily that mediates strand annealing and exchange reactions starting from DNA extremities. Redβ and RecA (the host recombinase) have different recombination mechanisms, substrates and rates [21]. The protein Gam inhibits the host RecBCD exonuclease activity thus allowing efficient rolling-circle replication [22]. Thus, Lambda blocks the host recombination, superseding it with its own encoded recombination machinery. Other phages use evolutionarily related (e.g. Erf in P22) or unrelated recombinases (Sak4 in HK620, related to RecA) as well as other inhibitors of the exonucleolytic activity of RecBCD (e.g. Abc2 in P22 or gp5.9 in T7) [23], [24].

Lambda and most of its mutants cannot produce concatemers from monomers using the host RecABCD pathway of homologous recombination because Gam inhibits RecBCD. When *gam* is experimentally inactivated, RecBCD prevents phage replication by degrading its genome. However, Lambda mutants that include a chromosomal sequence with the octamer Chi motif (GCTGGTGG) are viable [25]. This is because the destructive nuclease-helicase activity of RecBCD shifts to repair mode when it meets a Chi site by recruiting the RecA recombinase onto nascent Chi-containing ssDNA [26]. The single strand annealing protein RecA then promotes strand invasion and recombination. Chi sites are very abundant in *E. coli*, found in average every 5 kb, and much more frequently in the core genome than in recently acquired genes [27], [28]. Chi sites are absent from the wild-type genome of Lambda and this prevents the use of RecBCD to produce phage concatemers. The high frequency of Chi in the *E. coli* genome and its rarity in Lambda and phage T4 led to the hypothesis that Chi is implicated in the discrimination between self and non-self and that the RecBCD-Chi system also functions to protect the genome from mobile genetic elements [29]–[31].

Phage fitness depends on its ability to control its host and on what it pays for that in terms of genome space and production costs [32]. Phages encoding their own recombination mechanisms gain an advantage by using proteins that co-evolved with the phage for a long period of time and are thus adapted to it in terms of processivity and tolerance to sequence divergence. However, the expression of recombination functions takes up resources. Encoding these functions also takes up genome space. Lambdoids rarely exceed 60 kb in size and most are between 40 kb and 50 kb [10]. This suggests the existence of an optimal size beyond which further accretion of genetic material lowers the phage fitness. Loss of the recombination module might facilitate acquisition of other functions with higher adaptive value in certain ecological contexts as long as recombination functions can be found in the host and manipulated by the phage. Increase in phage genome size might also be costly because of the replication cost and because such genomes require larger virions [33]. Phages that manipulate host recombination functions do not pay these additional costs, but they must use machineries adapted to their hosts. These proteins might not fit optimally the phage requirements and may have a cost in terms of host range. On the other hand, these mechanisms are well adapted to the host genetic background. Here, we study phage recombination functions to understand how the dilemma between encoding and manipulating them shapes phage evolution.

## Results

### Chi sequences are abundant in lambdoids

We analyzed recombination functions encoded by lambdoid phages. These phages account for the majority *E. coli* prophages [10], and their recombination mechanisms have been thoroughly studied [18]. The classification of phages in the group of lambdoids is itself motivated by their ability to produce viable hybrids by recombination at high frequency. We identified Chi motifs in a set of 275 lambdoid phages of *Escherichia* and *Salmonella* (see Materials and Methods). We computed the expected number of the 8-nucleotide Chi motifs using four different statistical models: accounting for the frequency of nucleotides, tri-nucleotides, penta-nucleotides and hepta-nucleotides (see Materials and Methods). The different models gave concordant results (Table S1 and S2). We present the results for the tri-nucleotides model, which is the most adequate for the slightly degenerated Chi motif and the small genomes of phages (see Materials and Methods). We computed the number of Chi motifs observed/expected (O/E) ratio separately for each phage genome. Surprisingly, we found that, as a whole, lambdoids have more Chi motifs than expected (median O/E = 2.30, p<0.0001, Mann-Whitney test). In fact, most Lambda-like phages encode Chi motifs (85%), which are significantly more frequent in these phages than expected given sequence composition (median O/E = 2.43, p<0.0001, Mann-Whitney test). These results show that Chi sites are far from rare in phage genomes. In fact, they are much more abundant than expected given genome size and composition.

### Phage recombinases and RecBCD inhibitors shape the abundance of Chi sites

**Figure 2:**
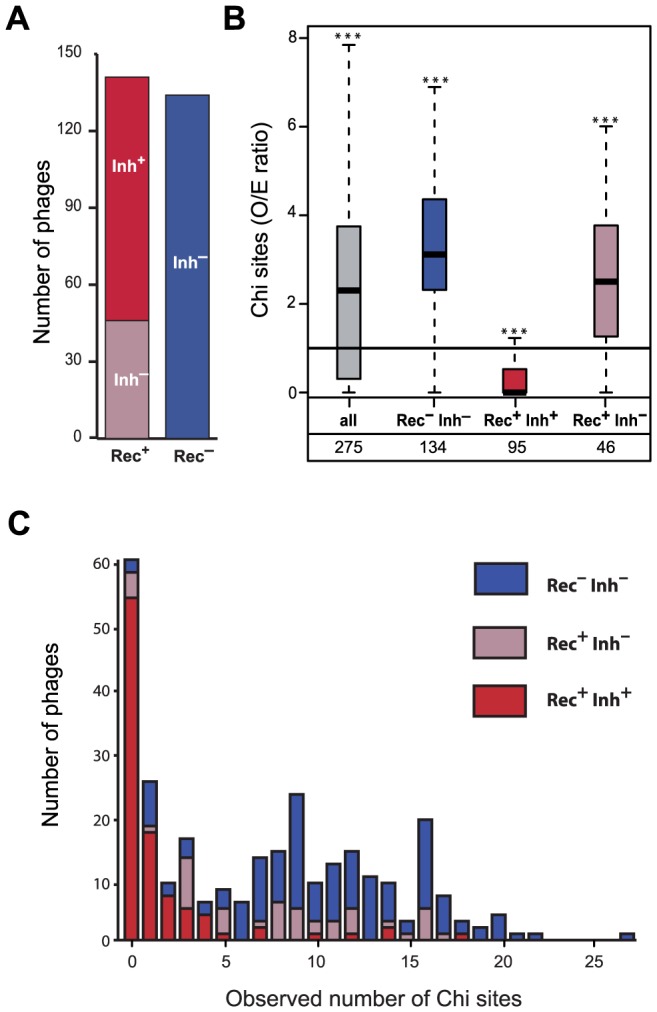
Association between the presence of phage recombination functions and the abundance of Chi sites. (*A*) Number of lambdoid phages encoding RecBCD inhibitors (Inh^+^/Inh^−^) and recombinases (Rec^+^/Rec^−^). (*B*) Distribution of the number of Chi sites observed/expected (O/E) ratios among lambdoid phages. Inh^+^ and Inh^−^ indicate the presence or the absence of a RecBCD inhibitor protein respectively. For each box, the lower and upper horizontal edges represent respectively the first and the third quartile. The middle bar of each box indicates the median value. The central vertical lines indicate the data range, with a maximal distance of 1.5 interquartile ranges (i.e. the distance between the first and third quartile values). The number of phages is indicated for every class. For each class, we tested if the median value of the O/E ratio among phages was significantly different from 1 with the Mann-Whitney test (* p<0.05, ** p<0.01, *** p<0.001). (*C*) Number of Chi sites among lambdoid phage genomes lacking a recombinase (Rec^−^) or encoding a recombinase with (Rec^+^ Inh^+^) or without (Rec^+^ Inh^−^) an inhibitor of RecBCD (Gam or Abc2). We found no phages Rec^−^ Inh^+^.

Phage genomes lacking recombinases require the host machinery to engage in homologous recombination. To test the hypothesis that this leads to selection for the presence of Chi sites to recruit RecBCD, we detected phage recombinases using protein clustering and profile-profile alignments (see Materials and Methods). We identified a recombinase in 141 genomes of lambdoids, i.e. approximately half of our dataset (Rec^+^ phages, 51%) (Figure 2A). Most of the identified recombinases (68%) are from the Redβ family, the one encoded by Lambda (Figure S1). Phage genomes lacking recombinases (Rec^−^ phages) display a significant over-representation of Chi sites (median O/E = 3.12, p<0.0001, Mann-Whitney test). These results are well in agreement with our hypothesis that phages lacking recombination functions select for the presence of Chi sites to recruit the host recombination machinery.

Phages encoding recombination functions but no RecBCD inhibitory functions could select for the presence of Chi motifs in their genomes to protect themselves from RecBCD exonucleolytic activity. To test this hypothesis, we searched for RecBCD inhibitors from the Gam and Abc2 families and identified 95 of these (see Materials and Methods). We found no single phage lacking a recombinase and encoding a RecBCD inhibitor. Red^−^Gam^+^ Lambda mutants are viable [19], showing that recombinases are not strictly required for phage replication when RecBCD is inhibited. On the other hand, RecBCD inactivation in the absence of phage recombinases has a very strong fitness cost in *E. coli* [34]. Cells where phages inhibit RecBCD without superseding it with their own recombinases lack tools to efficiently repair DNA double strand breaks. The fitness cost associated with this impairment might explain the lack of Rec^−^Inh^+^ phages in our dataset.

We found 95 phage genomes encoding a recombinase and a recombination inhibitor (Rec^+^Inh^+^). Among Rec^+^ phages, Inh^+^ phages display a significant under-representation of Chi sites (median O/E = 0, p<0.0001, Mann-Whitney test), whereas Inh^−^ over-represent Chi motifs (median O/E = 2.50, p<0.0001, same test) (Figure 2B and 2C). Importantly, while both Rec^+^Inh^−^ and Rec^−^ phages over-represent Chi, the latter show stronger over-representation (p<0.03, Wilcoxon test). Gam-like proteins inhibit RecBCD activity, whereas Abc2-like RecBCD inhibitors subvert RecBCD functions rendering them Chi-insensitive [35]. We tested if phages encoding Gam-like RecBCD inhibitors showed different degrees of avoidance of Chi motifs relative to those encoding Abc2-like RecBCD inhibitors. While there is a slightly stronger avoidance of Chi sites in Abc2 encoding phages (p = 0.030, Wilcoxon test), both Gam-like and Abc2-like RecBCD inhibitors are strongly associated with Chi motifs under-representation (median O/E of 0.30 and 0 respectively, both p<0.0001, Mann-Whitney tests). Hence, phages encoding recombinases but not RecBCD inhibitors have more Chi sites than expected, whereas phages with RecBCD inhibitors strongly avoid Chi sites. This suggests that Rec^+^Inh^−^ phages select for the presence of Chi sites, whereas Rec^+^Inh^+^ phages select for the absence of Chi sites. Phage Lambda is thus a typical representative of the Rec^+^Inh^+^ class of phages. These results show a strong link between the ability of a phage to inhibit the exonucleolytic activity of RecBCD and the presence or absence of Chi motifs.

### Chi motifs in phages and their hosts

We compared the frequency of Chi motifs in phages and their hosts. As observed previously [27], [28], Chi motifs are over-represented in the genomes of *E. coli* K12 and *S. enterica* Typhimurium (O/E = 2.29, p<0.0001 and O/E = 2.40, p<0.0001, Z score), and slightly more in the core genome of each species (resp. O/E = 2.36 and 2.38, both p<0.0001, same test, see Table S3 for the different models). The density of Chi sites in Rec^−^ phages is not significantly different from the host bacterial genome (0.2 Chi motifs/kb, p = 0.103, Mann-Whitney test). However, given their composition, Chi motifs are more over-represented in these phages than in the core genome of *E. coli* (p<0.0001, Mann-Whitney test). The over-representation of Chi sites in Rec^+^Inh^−^ phages is not significantly different from that of the core genome of *E. coli* (p = 0.30, same test, see Table S4 for the other models). These results suggest that phages lacking RecBCD inhibitors endure similar or even stronger selection for Chi motifs than their hosts.

**Figure 3:**
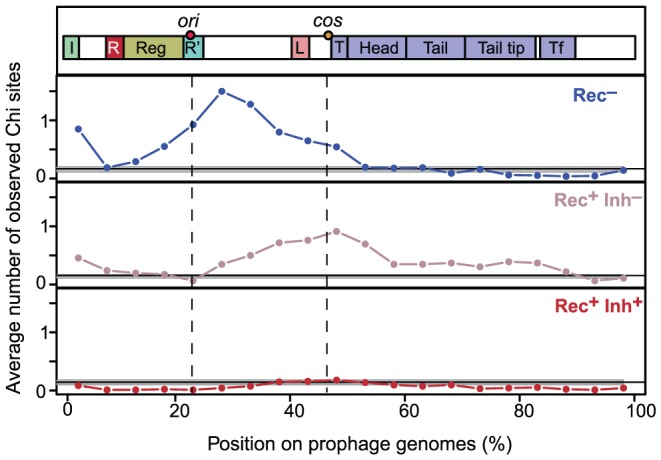
Distribution of Chi sites in lambdoid genomes. Lambdoid genomes (average length of 45 kb) were divided in 5% non-overlapping contiguous intervals (i.e. 2.25 kb). We plotted for each interval the average number of observed Chi motifs per phage. Phages were divided in three classes according to the encoded recombination functions: Rec^−^, Rec^+^Inh^−^ and Rec^+^Inh^+^. Integrases of the Tyrosine recombinase family were detected as in [10]. Each genome was polarized with the integrase on the left end (the few genomes lacking tyrosine recombinase integrases were discarded). A schematic representation of Lambda-like phages is given on top. Label “*ori*” indicates the median position of the homologs of Lambda gene O, which includes the origin of replication. The label “*cos*” indicates the median position of the start of the first terminase gene, which is where the *cos* site is located in Lambda.

Some of the phages in our dataset were sequenced from virions whereas others were identified from bacterial chromosomes. We tested if inaccurate delimitation of the latter might have affected the number of Chi motifs found in our dataset. The median O/E number of Chi sites was not significantly different between Rec^−^ phages and Rec^−^ prophages (resp. 4.76 and 3.08, p = 0.45, Wilcoxon test). This ratio was almost indistinguishable among Rec^+^Inh^−^ phages and prophages (resp. 2.42 and 2.52, p = 0.58, same test) and among Rec^+^Inh^+^ phages and prophages (both medians equal to 0, p = 0.84, same test). Thus, the trends we observe in the frequency of Chi motifs do not reflect biases associated with prophage detection. We also verified that Chi motifs in phages were not concentrated at the cargo region, typically at the edge of the element opposing the integrase [36]. Interestingly, we found that Chi motifs were concentrated far from this region and between the genes encoding the replication functions and the terminase, before the structural genes. In Lambda this corresponds to the region between the origin of replication (in gene O) and the *cos* site (before the terminase gene Nu1) where DNA is cut during packaging (Figure 3). The distribution of Chi sites along the chromosomes of Rec^+^Inh^−^ phages and Rec^−^ phages is different (p<0.0001, Kolmogorov-Smirnov test). Chi motifs are more concentrated near the origin of replication of Rec^−^ phages, and towards the *cos* site in Rec^+^Inh^−^ phages. These results show that Chi over-representation in lambdoids cannot result from inaccuracies in the delimitation of prophages and suggests a tight association between recombination, replication and packaging in phages.

### Phage recombinases promote gene repertoire diversification and mosaicism

**Figure 4:**
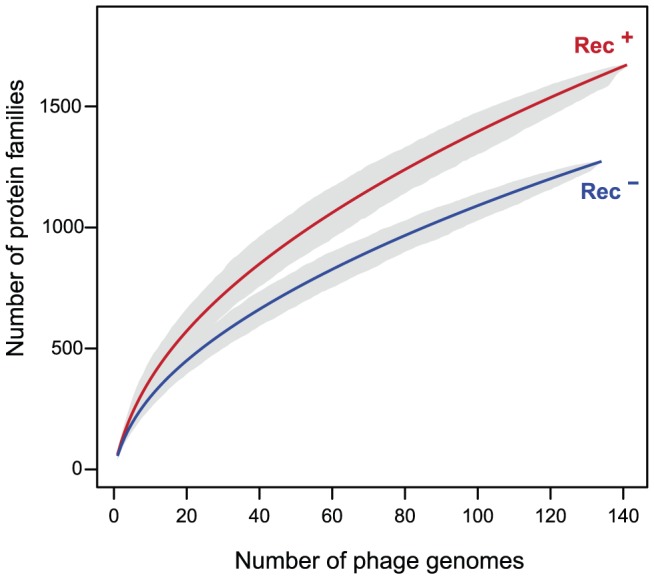
Pan genomes of the lambdoid phages encoding recombination functions (Rec^+^) are larger than those lacking them (Rec^−^). The pan genome size (y-axis) of each type of phage genome was computed for increasing numbers of genomes (x-axis). For each value of x we draw x genomes randomly and compute the pan genome. This is repeated 1000 times for each value of x to draw the 95% interval of confidence of the pan genome size (grey zone).

Recombination between different phages leads to genetic mosaicism and increases the diversity of gene repertoires. Redβ catalyzes recombination at higher rates and is more tolerant to sequence divergence than RecA [8]. We thus hypothesized that phages encoding recombination functions have more diverse gene repertoires. We built the pan genomes (i.e. the set of all different gene families) of Rec^+^ and Rec^−^ lambdoids (see Materials and Methods). The pan genome of Rec^+^ phages is systematically ∼22% larger than the pan genome of Rec^−^ phages for the same number of genomes (Figure 4). This effect could not be explained by genome size, which is indistinguishable between the two types of phages (average of 45 kb, p = 0.85, Wilcoxon test). Hence, the permissivity of phage recombinases might allow faster diversification of gene repertoires in phages encoding their own recombination functions.

**Figure 5:**
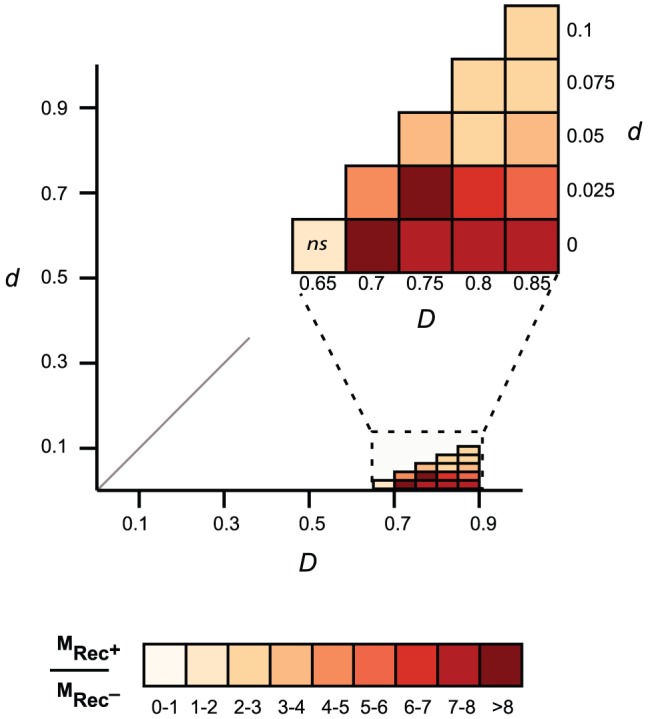
Comparison of gene mosaicism in 163 Lambda-like phages encoding (Rec^+^) or lacking (Rec^−^) recombinases. Mosaic genes are pairs of homologous genes with low evolutionary distances (low *d*) in phages with high evolutionary distances (high *D*). For each threshold T*_d_* and T*_D_*, we compared the frequency of mosaic genes of Rec^+^ phages (M_Rec_^+^) and Rec^−^ phages (M_Rec_^−^). The color scale gives the ratio of the frequency of mosaic genes between phages encoding and lacking recombination functions (M_Rec_^+^/M_Rec_^−^). Non-significant (p>0.05, Wilcoxon test) differences on the frequency of mosaic genes are indicated on the graph (*ns*).

We then tested the hypothesis that these phages are also more mosaic, i.e. exchange homologous genes at higher rates. For this, we identified highly similar homologous genes present in highly dissimilar phage genomes (see Materials and Methods). This is a conservative subset of the genes that have recently undergone recombination between distinct phages. We restricted the analysis to the 163 Lambda-like phages of *E. coli* since broader taxonomic groups share too few homologous proteins for reliable inference of distances between phages. We computed the distance matrices between homologous proteins (*d*) and between phages (*D*) and identified proteins for which *d* is small and *D* is large using a range of thresholds T*_d_* and T*_D_* (see Materials and Methods). The results consistently show that genes with low *d* encoded in phages of high *D* are very significantly over-represented in Rec^+^ phages (Figure 5). Rec^+^ phages have up to 8 times more such genes than Rec^−^ phages and this difference is particularly high for the most recent transfers (corresponding to the lowest values of *d*). We tested if these results could be explained by the nature of the genes undergoing recombination. We analyzed the functional categories of the transferred genes (Text S2), and found no significant differences between them and the remaining genes (p>0.1, χ^2^ test). We conclude that the higher mosaicism of phages encoding recombinases is independent of its phage gene repertoire size or content.

## Discussion

In this work we studied the presence in phage genomes of genes and DNA motifs involved in homologous recombination. We showed that some phages encode a large number of Chi motifs and are thus able to manipulate RecBCD. This provides certain advantages. First, for similar genome size, and thus capsid volume, this allows the genome to encode other potentially adaptive functions. Second, Chi sites protect from the exonucleolytic activity of RecBCD and thus also from restriction-modification systems [37]. Third, RecABCD recombination is less frequent between very divergent sequences and could lead to fewer non-viable hybrid genomes. Finally, Chi motifs being important for genome maintenance, the presence of Chi in prophages might stabilize the element and lower its fitness cost for the host. Prophages make up to 35% of the pan genome of *E. coli* and we have shown that they encode motifs associated with their local context in the bacterial chromosome [10]. Hence, prophages with Chi motifs might integrate more seamlessly in the host chromosome.

Some phages encode their own recombination machinery, inhibit the host's and avoid Chi motifs. Recombination autonomous to the host machinery also has some advantages. First, recombination machineries co-evolving with the phage should be better adapted to its specificities, e.g. in terms of recombination frequency, sequence composition or homology requirements. For example, RecT, a Redβ homolog from prophage Rac, shows preference for AT rich regions [38], which are typical of phages. Second, reduced dependence on the host's machinery might broaden the range of possible hosts. Even if the composition of the machinery of homologous recombination is similar in most non-intracellular γ-Proteobacteria [39], the Chi motifs of *E. coli* and *Haemophilus influenzae* show a number of differences [40]. Hence, phages relying on host recombination functions may be at a disadvantage in a new host encoding different Chi motifs. Third, Red recombination is more permissive to sequence divergence and this may enlarge the mutational landscape of the phage, accelerating its diversification.

The dilemma of encoding or manipulating host recombination functions may also impact ecological interactions between mobile genetic elements. For example, the protein Old encoded by phage P2 targets Redβ [41] and the AbiK system of *Lactococcus lactis* plasmids targets different phage recombinase families [42]. On the other hand, encoding autonomous recombination functions may render the phage less susceptible to mobile elements that compete to manipulate host recombination. During co-infection, phages encoding RecBCD inhibitors might therefore have an important advantage over Chi-dependent phages by reducing the number of concatemeric chromosomes they can produce.

The chromosomes of *E. coli* strains are packed with prophages, some of which contribute to important adaptive functions. Different temperate phages may recombine in the bacterial cell. These cells may thus work as ‘phage factories’, releasing a wide variety of recombinant phages in the environment [43]. We have shown that phages carrying their own recombination functions have more mosaic genomes and larger pan genomes. The gene repertoires of bacteria are in constant genetic flux partly due to the action of phage transduction. For example, the recent epidemic of *E. coli* in Germany was the direct consequence of toxins encoded by prophages [44]. Adaptive associations between phage and bacteria can be very complex, e.g. a bacterial endosymbiont prophage protects aphids from parasitoid wasps [45]. As mentioned above, recombination is also important in the context of the ongoing arms races between phages and their hosts. Hence, the way phages recombine may impact their rates of diversification, but also those of their bacterial hosts.

The absence of Chi in phage Lambda was instrumental to the discovery of the function of this motif [46]. It was also interpreted as lack of selection for the presence of Chi sites in phages carrying their own recombination systems [29]. Here, we showed that contrary to common belief Chi sites are very abundant in most phages. Yet, these results also put forward a puzzling observation. RecBCD inhibitors render Chi sites useless either by blocking the activity of the protein or by rendering it insensitive to Chi. Hence, phages encoding RecBCD inhibitors should have a number of Chi sites close to the random expectation given sequence length and composition. Surprisingly, we show that these phages strongly avoid Chi sites, i.e. they have fewer sites than expected. Chi is thus selected *against* in phages encoding RecBCD inhibitors and *for* in the other phages. This suggests that carrying simultaneously Chi sites and RecBCD inhibitors is deleterious for the phage. We have no good explanation for these intriguing results at the moment. One might speculate that Chi sites affect the efficiency of RecBCD inhibitors, but this is at odds with the observation that the *E. coli* chromosome is packed with Chi motifs. Chi avoidance might be related to the chromosomal context of the prophage and how it affects chromosome maintenance processes, e.g. selection for recombination outside the prophage element to avoid chromosomal rearrangements [47]. But this would suggest that Chi are deleterious to integrative elements, which seems at odds with the large number of Chi sites found in the majority of prophages. Understanding selection against Chi sites will require further experimental work.

We showed that Chi sites in phages are concentrated between the origin of replication (especially in Rec^−^ phages) and the packaging sites (especially in Rec^+^Inh^−^ phages). Naturally, the origin and *cos* (or *pac*) sites are unknown for the majority of phages and this result must be interpreted with care since it assumes that among lambdoids these positions are relatively unchanged. Nevertheless, the high density of Chi in the origin and packaging site regions, and the differences between the two regions in terms of phage recombination repertoires suggest some sort of selection for Chi sites in these locations. In fact, the very high frequency of Chi motifs in such a small region, up to three times the density in the *E. coli* core genome, might explain why this region is unusually variable among lambdoid genomes (the *nin* region [7], [48]). The association between replication and recombination is pervasive in cellular organisms [1] and phages lacking recombinases might thus select for Chi sites near the origin of replication to process stalled replication forks. In phages encoding a recombinase able to process stalled replication forks, Chi sites might be more important for protection of free DNA ends from degradation by RecBCD than for its recruitment for recombination, explaining the fewer Chi sites and their location close to the packaging site in these phages. Hence, the study of the roles of Chi sites in phages might enlighten further functional associations between recombination, phage replication and packaging.

To check on the generality of our observations, we made some preliminary analyses of non-lambdoid *E. coli* phages in GenBank (Table S5 and Text S3). These analyses are hampered by the small dataset for each phage family and the lack of available information on the mechanisms of recombination in most genera. Yet, we could verify that phages requiring concatemers for packaging over-represent Chi motifs relative to phages able to encapsidate monomers (p<0.0001, Wilcoxon test). The two phage genera requiring concatemers for packaging and lacking recombinases (T5-like and P1-like) exhibit the strongest over-representation of Chi motifs (Table S5). The Chi abundance in P1-like phages shows that Chi sites can also be abundant in non-integrative temperate phages. T5 is a virulent phage showing that Chi over-representation is not limited to temperate phages. The reliable identification of presence or absence of specific RecBCD inhibitors is difficult in non-lambdoids because of the phage diversity and the tendency of RecBCD inhibitors to be small family-specific and fast-evolving proteins. Yet, these results suggest that Chi-dependent recombination might be widespread among phages packaging concatemeric DNA, for which recombination is important, even among virulent phages and non-integrative temperate phages.

Dilemmas between manipulation and supersession of host functions are probably common in viruses. For example, some phages encode tRNAs to complement the host's repertoire [49] and some filamentous phages encode their own secretion apparatus whereas others manipulate their host's secretion systems [50]. In fact, pathogenic bacteria or protozoa manipulating host functions might also face similar trade-offs [51]. Understanding why different parasites evolved to manipulate host functions or to encode their own, can provide important clues on their mechanisms of virulence and, as we showed, of their evolvability.

## Materials and Methods

### Genome data

The complete genomes of *Escherichia* (47 *E. coli*, 1 *E. fergusonii*) and *Salmonella* (20 *S. enterica* and 1 *S. bongori*) were downloaded from NCBI RefSeq (ftp://ftp.ncbi.nih.gov/genomes/). We analyzed a total of 275 phages including 38 lambdoid phages infecting enterobacteria (downloaded from RefSeq) and 237 long (>30 kb) non-redundant lambdoid prophages from the genomes of the abovementioned species identified in [10] with different mobile element detections [52]–[55] (see also Text S1 and Table S6). Among the 131 non-lambdoid genomes, 80 phage genomes of the *Caudovirales* order (69 virulent and 11 temperate) were downloaded from RefSeq (when classified in a genus defined by the ICTV). And 51 non-lambdoid prophages were identified in [10] with the same criteria (>30 kb and non-redundant).

### Core and pan genomes

The core genomes of *E. coli* and *S. enterica* were computed as described previously [10]. The pan genomes were computed from the 141 Rec^+^ lambdoid phages (9108 proteins), the 134 Rec^−^ lambdoid phages (7554 proteins), and also the 163 Lambda-like phages of *E. coli* (9856 proteins). Homologous proteins were defined as pairs of proteins with more than 40% sequence similarity, computed using a Needleman-Wunsch end gap free alignment algorithm with the BLOSUM62 matrix, and with less than 50% of difference in length. Protein families were built from the pairwise analyses by transitivity, i.e. a protein is included in the family if it shares a relation of homology to a protein already in the family. The pan genome is the set of all different protein families. We excluded Genbank entries NC_004913, NC_004914 and NC_003525 from this analysis because their annotations over-predict the number of genes (nearly three times more genes per kilobase than the average lambdoid phage).

### Identification of recombinases

We compared all lambdoid phage proteins to each other using blastp (*e*-value<0.001). The resulting blast bit score was used to cluster the proteins with MCL [56]. After testing the MCL inflation parameters in the range [1.2 to 5.0], we used I = 3.0 because it was the smallest that produced protein clusters where all proteins of each cluster could be analyzed in a single multiple alignment. A total of 1812 protein clusters were obtained for the 16662 proteins analyzed. We aligned the proteins of each cluster with MUSCLE v3.6 [57] and built protein profiles with the HH-suite v2.0.9 [58]. The protein profiles of recombinases were initially found by comparison with published profiles [24] using HHsearch (profile-profile comparison, p>95% in local and global alignments and >50% of profile coverage). We identified initially a subset of 14 protein clusters. To exclude helicases with ATPase domains from recombinases [24] we also made profile-profile comparisons with PFAM-A profiles (downloaded the 11/25/2011) using HHsearch (same parameters). We excluded the clusters matching PFAM-A profiles annotated as helicases (e.g. DnaB, helicase-ATPase domain, DEAD/DEAH box helicase, PIF1-like helicase), producing a final set of 8 protein clusters of recombinases. This corresponds to 141 proteins found in 141 lambdoids. Our procedure was able to find all of the recombinases previously identified in lambdoid phages of enterobacteria [24].

### Identification of RecBCD inhibitors

We searched lambdoid phage genomes for hits of PFAM profiles of Gam (PF06064) and Abc2 (PF11043) proteins using HMMER v3.0 (*c*-value<10^−5^) [59]. A total of 95 RecBCD inhibitors were detected: 56 Gam and 39 Abc2 proteins. The families of RecBCD inhibitors from T7 (gp5.9, NP_041987), *Enterococcus* phage BC-611 (ORF41, BAM44931), *Clostridium* phage phi8074-B1 (phi8074-B1_00044, AFC61976) and the DNA end protector from T4 (gp2, NP_049754), have not been described among enterobacterial temperate phages. Indeed, we found no significant BLASTP hits (at a threshold of *e*-value<0.001) to these proteins in our dataset of lambdoid phages.

### Detection of Chi motifs

We used R'MES v3.1.0 to search the non-degenerated Chi motif 5′GCTGGTGG3′ and to compute significance of Z scores [60]. We computed the number of expected and observed Chi motifs accounting for the oligonucleotide composition separately for each genome. This was done to avoid putting together different phage genomes, which differ extensively in terms of nucleotide composition [61]. Four statistical models were analyzed for each genome. 1) The simplest model (M0) accounts only for nucleotide composition. 2) The M2 model accounts for the composition in tri-nucleotides. 3) The M4 model accounts for the composition in penta-nucleotides. 4) The maximal model (M6) accounts for the frequency of the maximal sub-strings of Chi motifs, i.e. hepta-nucleotides. The four models produced concordant statistics (Table S1). The M0 model is a poor predictor of random usage of large oligonucleotides because these are also affected by selection on other smaller oligonucleotides such as codons [62]. Phage genomes are small (<50 kb on average) and the Chi motif is slightly degenerated [63]. These two traits hinder the statistical power of the M6 and M4 models. Therefore we show in the text the results of the M2 model. The statistical significance of Chi sites over or under-representation in a given set of phages was computed using the Mann-Whitney test. Chi sites over-representation per genome was assessed by the Z score computed with R'MES. We computed all models under the compound Poisson approximation that is more adequate for low counts [60].

### Analysis of gene mosaicism

We initially aimed at using classical phylogenetic approaches to identify recombination events. Unfortunately, no proteins are ubiquitous to the whole set of 163 Lambda-like phages of *E. coli*. We therefore designed a method to find highly similar pairs of homologous proteins in two otherwise distantly related phages, which are likely the result of recent recombination events (mosaic genes). This approach resembles closely that of [64]. First, we constructed the multiple alignment of each protein family of the pan genome of Lambda-like phages of *E. coli* with MUSCLE v3.6 [57]. Second, we extracted the informative positions in the alignments using BMGE with the BLOSUM30 matrix [65]. The 19 (4%) protein families with trimmed alignments shorter than 50 sites were excluded due to the lack of phylogenetic signal. Third, we computed the protein distances (*d_i,j_^F^*) of each pair of homologous proteins between two phages *i* and *j* in every protein family using TREE-PUZZLE v5.2 [66]. The distance matrix was computed using maximum likelihood under automatic estimation of the best substitution model and a Γ(8) correction for rate heterogeneity. Fourth, the distance matrix between phages *D_i,j_* was defined as the mean value of *d_i,j_* for the orthologs shared by each pair of phages *i* and *j*. For each pair of phages, orthologous proteins were defined as unique reciprocal best hits with more than 40% similarity in amino acid sequence and less than 50% of difference in protein length. Finally, mosaic genes were identified as the ones encoding highly similar homologous proteins in highly dissimilar genomes for different thresholds T*_d_* and T*_D_*. More precisely, a pair of homologous genes between two phages *i* and *j* was regarded as mosaic if the encoded proteins were closey related (*d_i_*_j_<T*_d_*) and the two phages were distantly related (*D_i,j_*>T*_D_*). The different thresholds tested T*_d_* and T*_D_* showed qualitatively similar results. We did not analyze recombination events in genes encoding recombination functions, because they are absent from Rec^−^ phages. We also ignored transposable elements, because they are self-mobilizable.

## Supporting Information

Figure S1 Recombinase families identified in lambdoid phages. Recombinases were identified by profile-profile comparisons with HHsearch (see Materials and Methods). Most of the identified recombinases belong to the Rad52 superfamily (Redβ, Erf and Sak). Sak4 recombinases are part of the Rad51 superfamily and are remote homologs of RecA [24]. Gp2.5 represents the last superfamily of phage recombinases and is found much more frequently in virulent phages [24].
(EPS)

Table S1 Chi sites Observed/Expected ratio for lambdoid phages and their bacterial hosts computed with models M0, M4 and M6. The expected number of Chi sites has been determined with three additional models: M6, M4 and M0. For each category, we tested if the ratio O/E of Chi composition in the set of phages was significantly different from random expectation (O/E = 1) with the Mann-Whitney test.
(XLS)

Table S2 Chi sites Z score statistics for lambdoid phages and their bacterial hosts. The expected number of Chi sites has been determined with the M2 model. For each category, we tested if the Z score of Chi composition in the set of phages was significantly different from random expectation (Z = 0) with the Mann-Whitney test. The “Skew” column indicates if the phage category over-represents (+) or under-represents (−) Chi sites.
(XLS)

Table S3 Chi sites Observed/Expected ratio for *E. coli* and *S. enterica* with models M0, M4 and M6. The expected number of Chi sites has been determined with three additional models: M6, M4 and M0. For each core or complete genome, we tested if the Chi composition was significantly different from random expectation with the Z score. The analysis was run on *E. coli* K12 MG1655 and *S. enterica* Typhimurim LT2 genomes respectively.
(XLS)

Table S4 Comparison of the Chi sites Observed/Expected ratio of *E. coli* lambdoid phages and all lambdoid phages to the Chi sites Observed/Expected ratio of *E. coli* core genome with models M0, M4 and M6. The median value “M” of the Chi sites Oberved/Expected ratio is given for lambdoid coliphages and for all lambdoid phages for each category. For each category and model, we tested if the Chi composition was significantly different from the Chi composition of *E. coli*'s core genome with the Mann-Whitney test. The analysis has been done on the core genes of *E. coli* K12 MG1655.
(XLS)

Table S5 Chi sites Observed/Expected ratio and Z scores for different genera of phages and prophages infecting enterobacteria. We used the non-lambdoid phage genera of the *Caudovirales* order defined by the ICTV. Prophages were identified and classified as in [10]. Phage's life style, i.e. virulent (v) and temperate (t) is indicated in the “Type” column. The type of DNA substrate used for packaging, i.e. concatemeric (C) of monomeric (M) is indicated in the “Packaging” column.
(XLS)

Table S6 Description of lambdoid phages and prophages. For each phage (ph) or prophage (pro) used for the analysis, the RefSeq ID of the host genome is given for prophages and the RefSed ID is given for phage genomes directly. The recombinase family (Redβ, ERF, Sak, Sak4 and Gp2.5) is indicated in the “Rec” column. The type of RecBCD inhibitor (Gam or Abc2) is indicated in the “Inh” column. The expected number of Chi sites is given for the tri-nucleotides model (M2). For each genome, Chi composition significantly different from random expectation is given by the “*pvalue* (Z score)” column. The GC content of each phage or prophage genome is given in the “GC” column.
(XLS)

Text S1 Identification and classification of prophages.
(DOC)

Text S2 Function assignment.
(DOC)

Text S3 Detection of Chi sites in non-lambdoid phages.
(DOC)
