# Supplementary material for: Manipulating or Superseding Host Recombination Functions: A Dilemma That Shapes Phage Evolvability
Source: PLoS Genet. 2013 Sep 26;9(9):e1003825. doi: 10.1371/journal.pgen.1003825 (PMC3784561; doi:10.1371/journal.pgen.1003825)
Supplement: Text S1 — Identification and classification of prophages. (DOC) [file pgen.1003825.s008.doc]

Text S1. Identification and classification of prophages.

**Identification of prophages**. Prophages were detected as in [10]. i) The initial detection process used three prophage-detection programs: Phage Finder [52], PHAST [53] and Prophinder [54]. These programs combine sequence comparisons to known phage or prophage genes, comparisons to known bacterial genes, identification of tRNA genes, dinucleotide frequency analysis and identification of attachment sites. ii) We then removed putative prophages with a large number of Insertion Sequences (>25% of the predicted genes). IS elements were detected as in [55]. iii) In the third step, prophage borders and the few tandem were manually curated using gene annotation, hits to PFAM and the definition of core/pan bacterial genomes. iv) From the resulting 500 prophages we removed the shorter than 30 kb to avoid partially degraded prophages. The threshold of 30kb has been chosen as it represents the minimum size range of a functional temperate - and non-satellite - phage genome infecting enterobacteria within our *Caudovirales* phage dataset (*Salmonella* phage PsP3). v) Finally, pairs of prophages with a repertoire relatedness score over 0.9 (see [10]), were considered as redundant and only the longer prophage was kept for further analysis. A total of 301 prophages were thus obtained and correspond to the non-redundant long dataset (NRlong) of [10].

**Phage classification**. Using taxonomic information from the ICTV and the literature we were able to classify most of the detected prophages (i.e. to attribute them an order, a family, a genus and the membership to the lambdoid group) [10]. The majority of temperate phages were attributed to the lambdoid group which contains the following genera/groups: Lambda-like, P22-like, SfV-like, Stx-like, N15-like and unclassified lambdoids. The non-lambdoid temperate phages were classified in P2-like, Mu-like, P1-like, Epsilon15-like and unclassified non-lambdoids genera/groups. The rest of the analysis focuses on these 237 lambdoid prophages thus classified and the 38 lambdoid phages downloaded from RefSeq.
