# Supplementary material for: Manipulating or Superseding Host Recombination Functions: A Dilemma That Shapes Phage Evolvability
Source: PLoS Genet. 2013 Sep 26;9(9):e1003825. doi: 10.1371/journal.pgen.1003825 (PMC3784561; doi:10.1371/journal.pgen.1003825)
Supplement: Text S2 — Function assignment. (DOC) [file pgen.1003825.s009.doc]

Text S2. Function assignment.

We used HHsearch to search for genes in phages matching PFAM-A protein profiles (parameters: p>95% in local and global alignments and >50% of profile coverage). Phage functions were assigned to the protein clusters using this information. Functions that are specific of phage were automatically attributed to the protein clusters from PFAM-A profile descriptions. The following functional classes were defined: "integrase", "excisionase", "resolvase", "transcription regulator", "replication protein", "lysis", "terminase", "portal protein", "packaging protein", "head protein", "head tail connector", "tail protein", "tail fiber protein", "methyl tranferase", "virulence protein" and "transposase". Around 63% of protein clusters didn't display a significant match with any of the functions listed before and were classified as "unknown".
