# Supplementary material for: Manipulating or Superseding Host Recombination Functions: A Dilemma That Shapes Phage Evolvability
Source: PLoS Genet. 2013 Sep 26;9(9):e1003825. doi: 10.1371/journal.pgen.1003825 (PMC3784561; doi:10.1371/journal.pgen.1003825)
Supplement: Text S3 — Detection of Chi sites in non-lambdoid phages. (DOC) [file pgen.1003825.s010.doc]

**Text S3. Detection of Chi sites in non-lambdoid phages**

We computed the Chi sites O/E ratio for the genomes of 131 non-lambdoid phages and prophages, including temperate and virulent phage genera infecting enterobacteria. The details of the results are given table S5. We estimated the over- or under-representation of Chi sites of these genomes individually by using the Z score statistics with the tri-nucleotides (M2) model. Two genera significantly and consistently over-represent Chi sites: the non-integrative temperate P1-like phages and the virulent T5-like phages. To the best of our knowledge and according to our detection of recombinases and RecBCD inhibitors, these phages don't encode recombinases. Moreover, these elements only package their genomes from concatemeric DNA. It is therefore likely that they manipulate the host recombination functions through the presence of Chi sites in their genome.

The other phages show less clear patterns of over or under-represent Chi motifs. The small sample sizes preclude taking very solid conclusions from these trends. For example some groups have no single Chi motif, but even this extreme under-representation is not statistically significant because of the small sample size. This is the case of P2-like, P4-like and Mu-like phages. Interestingly, all these phages are able to package monomeric DNA and therefore might be under weaker selection for using the host recombination functions. T7-like and Epsilon15-like phages seem to slightly over-represent Chi sites and both genera were found to encode a recombinase (Gp2.5 and Redβ respectively). We could not find a RecBCD inhibitor in Epsilon15-like phages. A minority (32%) of T7-like phages encodes an homolog of the RecBCD inhibitor protein Gp5.9, but the sample size precludes a comparison between Inh^+^ and Inh^–^ phages in this case. It is therefore likely that T7-like and Epsilon15-like phages use Chi sites as a protection from the host RecBCD exonuclease like Rec^+^Inh^–^ lambdoids. But for these and other clades, the identification of clear patterns will require a much larger sample size and a better knowledge of their RecBCD inhibitors.
